# Pregnancy among Women Undergoing Intrauterine Insemination in a Centre

**DOI:** 10.31729/jnma.8234

**Published:** 2023-08-31

**Authors:** Sanu Maiya Shrestha Pradhan, Anita Karki, Amit Khanal, Namuna Ghimire, Renee Pradhan

**Affiliations:** 1 Creators IVF Nepal Pvt. Ltd., Satdobato, Lalitpur, Nepal

**Keywords:** *artificial insemination*, *Nepal*, *pregnancy rate*

## Abstract

**Introduction:**

Intrauterine insemination is often performed as the first-line treatment option in many subfertility cases. Successful intrauterine insemination treatment among indicated couples helps to reduce unnecessary in-vitro fertilisation treatment. Although widely practised, the data on pregnancy after intrauterine insemination treatment is limited. The aim of this study was to find out the prevalence of pregnancy among women undergoing intrauterine insemination in a centre.

**Methods:**

A descriptive cross-sectional study was conducted at a centre among women undergoing intrauterine insemination. Data from 15 May 2017 to 15 April 2021 were collected between 16 June 2022 to 1 July 2022 from the hospital records. Ethical approval was taken from the Nepal Health Research Council. Systematic sampling technique was applied to select the appropriate sample. The point estimate was calculated at a 95% Confidence Interval.

**Results:**

Among 225 women undergoing intrauterine insemination, pregnancy was achieved in 23 (10.22%) (6.26-14.18, 95% Confidence Interval). The mean age of pregnant women was 29.17±3.34 years and the mean duration of infertility was 3.93±2.90 years. More than half of the pregnant women 13 (56.52%) had undergone insemination with the husband's semen whereas the remaining 10 women (43.48%) had undergone insemination with donor semen.

**Conclusions:**

The prevalence of pregnancy among women undergoing intrauterine insemination was similar to other studies done in similar settings.

## INTRODUCTION

Among medically assisted reproduction (MAR) techniques, intrauterine insemination (IUI) is often performed as the first-line treatment option in many subfertility cases.^1^ In IUI, the partner's or donor's sperm is processed by physicochemical methods to obtain highly concentrated motile sperm, which is then inseminated into the woman's uterus through the cervix using a small catheter.^2^ It is a safe, relatively inexpensive, and non-invasive procedure. Most countries have a lack of IUI registration or incorporation of IUI procedures reported within In Vitro Fertilisation (IVF) registries.^3^

Successful IUI treatment among indicated couples helps to reduce unnecessary in-vitro fertilisation (IVF) treatment. Although widely practised in Nepal, data on the pregnancy rate following IUI treatment is limited.

The aim of this study was to find out the prevalence of pregnancy among women undergoing intrauterine insemination in a centre.

## METHODS

A descriptive cross-sectional study was conducted at Creator's IVF Nepal Pvt. Ltd. (CIVF), Satdobato, Lalitpur Shrestha Pradhan et al. Pregnancy among Women Undergoing Intrauterine Insemination in a Centre Nepal. Data from 15 May 2017 to 15 April 2021 were collected between 16 June 2022 to 1 July 2022 from the hospital records. Ethical approval was taken from the Nepal Health Research Council (Reference number: 4158). All the women who underwent IUI during the study period were included in the study. IUI cycles where ovulation was not induced by human chorionic gonadotropin (hCG) and had undergone spontaneous rupture were excluded as the data on follicle size for these IUI cycles could not be recorded. Women with incomplete medical records were excluded from the study. Systematic sampling technique was applied to select the appropriate sample.

The sample size was calculated using the following formula:


$$\begin{array}{ll} \text{n} & ={\text{Z}}^{2}\times \frac{\text{p}\times \text{q}}{{\text{e}}^{\text{2}}}\\ & =1.96^{2}\times \frac{0.157\times 0.843}{0.05^{2}}\\ & =204 \end{array}$$

Where,

n = minimum required sample size
Z = 1.96 at 95% Confidence Interval (CI)
p = prevalence taken from the previous study as, 15.7%^4^
q = 1-p
e = margin of error, 5%

The minimum required sample size was calculated to be 204.

For systematic random sampling, sampling interval 'k' was calculated using the following formula:


$$\begin{array}{l} \text{k=}\frac{\text{N}}{\text{n}}\\ =\frac{807}{208}\\ =4 \end{array}$$

Where,

k = sampling interval
N = estimated total population during the study period
n = minimum required sample size

Using computer generated random number '4' was selected as starting number and after that every 4^th^ number was selected till minimum sample size was reached.

The age of the woman, age of husband, duration of infertility in years, type of infertility, menstrual cycle, indication for IUI, ovarian stimulation regime, mean diameter of dominant follicle on trigger day, endo-thickness in last scan, type of insemination, number of inseminations per cycle and post wash sperm concentration were the study variables. The outcome variable was the pregnancy rate (PR) per couple.

Data were entered and analysed using IBM SPSS version 20.0. The point estimate was calculated at a 95% Confidence Interval.

## RESULTS

Among 225 women who underwent IUI at the fertility centre, pregnancy was achieved in 23 (10.22%) (6.2614.18, 95% CI). The mean age of pregnant women was 29.17±3.34 years. The average duration of infertility was 3.93±2.90 years. Among all pregnant women, 13 (56.52%) had undergone insemination with the husband's semen whereas the remaining 10 (43.48%) had undergone insemination with donor semen (Table 1).

**Table 1:** Clinical and insemination-related parameters among pregnant couples (n = 23).

Characteristics	n (%)
**Menstrual cycle**	
Regular	14 (60.87)
Irregular	9 (39.13)
**Type of infertility**	
Primary	17 (73.91)
Secondary	6 (26.09)
**Indication for IUI**	
Male factor	11 (47.83)
Unexplained	12 (B2.17)
**Ovarian stimulation regime**	
Natural cycle	2 (8.70)
Stimulated cycle	21 (91.30)
**Type of insemination**	
IUI-D	10 (43.48)
IUI-H	13 (B6.B2)
**Number of inseminations per cycle**	
Single sitting IUI	4 (17.39)
Double sitting IUI	19 (82.61)
**Post wash sperm concentration**	
≥15 mil/ml	20 (86.96)
<15 mil/ml	3 (13.04)

Majority of the women 14 (60.87%) were in <30 years age group (Table 2).

**Table 2:** Distribution of age group among pregnant women (n = 23).

Age group of women (in years)	n (%)
<30	14 (60.87)
30-34	8 (34.78)
35-39	1(4.35)
>40	-

## DISCUSSION

In our study, the prevalence of pregnancy among women undergoing intrauterine insemination was found to be 10.22%. This finding is lower than a similar study conducted in Nepal which reported a prevalence of 15.7% among 380 couples.^4^ The difference in the prevalence of pregnancy can be attributed to the different clinical characteristics of the couples undergoing IUI.^4^ In a similar study conducted in southeast Nigeria, the prevalence as confirmed by ultrasound was 10.4% and another study reported 306 pregnancies out of 3012 IUI cycles i.e. 10.2% which were similar to our study.^5,6^

In our study, the mean age of women in pregnant cycles was 29.17±3.34 years. Among all women who achieved pregnancy, the majority 40 (60.87%) belonged to the age group 30 and lower. This finding is similar to previous literature in which higher PR was seen among younger women.^2,7-10^ This can be attributed to natural depletion in oocyte quality and quantity with the increasing age of women.^11^ As our study also suggests more pregnant women were of lower age groups hence clinicians must carefully evaluate the couple's clinical profile before considering IUI among older women.

In our study, more than half of pregnant couples had undergone insemination with the husband's semen 13 (56.52%) and 10 (43.48%) had undergone insemination with donor semen. The International Committee for Monitoring Assisted Reproductive (ICMART) 2012 reported that the pregnancy rate after insemination with the husband's semen was 11.9%. On the other hand, the PR following insemination with donor semen reported by ICMART in 2012 i.e. 16.6%.^12^ The existing literature shows that satisfactory PR can be achieved by IUI with donor sperm. Hence, IUI-D should be considered a cost-effective fertility treatment for couples with azoospermia or couples with severe male factor infertility who cannot afford IVF.

In our study 19 (82.61%) women had undergone double sitting IUI whereas 4 (17.39%) women underwent single sitting IUI cycles. A growing body of evidence suggests that double insemination does not yield better PR as compared to single insemination.^3,6^ Further, a randomised control trial also failed to show significant improvement in the live birth rate with double-sitting IUI as compared to single-sitting IUI.^13^ In this regard, future prospective studies that compare the effectiveness of double-sitting IUI and single-sitting IUI for different infertility aetiology and treatment regimens are recommended.

With respect to sperm characteristics, in our study 20 (86.96%) of the women had a post-wash sperm concentration of ≥15 mil/ml whereas in 3 (13.04%) the post-wash sperm concentration of <15 mil/ml was obtained. In another study, it was reported that sperm concentration of >30 mil/ml increased the likelihood of pregnancy by 5 times as compared to sperm concentration of less than 5 mil/ml.^14^ However, there are some studies that contradict this evidence.^10,15^

The major limitation of our study is that the outcome measure is PR and not clinical pregnancy rate (CPR) or live birth rate (LBR). This was a single centre-based study so the findings may not be generalizable to all the couples undergoing IUI and must be interpreted with caution.

## CONCLUSIONS

In our study, the prevalence of pregnancy among couples undergoing intrauterine insemination was similar to other studies done in similar settings.
